# Perceived Overqualification at Work: Implications for Voice Toward Peers and Creative Performance

**DOI:** 10.3389/fpsyg.2022.835204

**Published:** 2022-04-27

**Authors:** Yi Li, Yan Li, Peilin Yang, Man Zhang

**Affiliations:** ^1^School of Management, Shanghai University, Shanghai, China; ^2^Party School of Anhui Provincial Committee of C.P.C., Hefei, China

**Keywords:** perceived overqualification, voice toward peers, peer-group perceived overqualification, creative performance, conservation of resources theory

## Abstract

Drawing on the conservation of resource theory, we examined the effect of perceived overqualification on the creative performance *via* voice toward peers, and how the peer group perceived overqualification moderates the relationship between perceived overqualification and creative performance. We tested this proposal using three waves of lagged data collected from 206 company employees in Shandong Province, China. The results revealed that peer group perceived overqualification moderated the indirect effects of perceived overqualification on creative performance such that there was positive indirect effect *via* voice toward peers when peer group perceived overqualification is high and negative indirect effect *via* voice toward peers when peer group perceived overqualification is low. The implications, limitations, and future directions of these findings were discussed.

## Introduction

As a result of the economic recession and popularization of higher education, the phenomenon of overqualification is becoming more and more common across the world. Perceived overqualification reflects the extent to which employees consider themselves possessing more education, experience, or skills than required by their jobs (Maynard et al., 2006). According to the organization for economic cooperation and development (OECD), 14.7% of employees feel overqualified for their jobs in European Union, with comparable and even higher rates of perceived overqualification in other countries, 15.6% in the United States, 30.5% in Chile and 23.7% in Greece (Organisation for Economic Co-operation and Development (OECD), 2017).

To date, a large number of literature have shown that perceived overqualification is related to a series of negative outcomes, such as less positive work attitude and lower happiness (Harari et al., 2017), less organizational citizenship behavior (Luksyte et al., 2020), and more counterproductive work behavior (Kim et al., 2019). At the same time, there are new evidence that perceived overqualification may be associated with more positive results. For example, overqualified employees may exhibit higher task performance and more proactive behavior (Zhang et al., 2016; Lee et al., 2021). However, empirical evidence of its link with positive outcomes is still lacking and the area is under-explored (Dar and Rahman, 2020). Given that knowledge and education are important predictors of creative performance (Willey Ramos et al., 2018), employees with excess qualifications may perform better in creative activities. Creative performance refers to the extent to which employees generate novel and useful ideas regarding procedures and processes at work (De Stobbeleir et al., 2011), which has become increasingly crucial to an organization’s survival and competitive advantage in a rapidly changing environment (Carmeli et al., 2013). So, when and how perceived overqualification affects employee creative performance?

Voice behavior is defined as the behavior of proactively challenging the status quo and making constructive suggestions (Vandyne et al., 1995), which is a key driver to promote creative performance. By identifying opportunities for improvement, participating in voice behavior can provide cognitive resources for creative performance (Grant, 2013). Studies have shown that voice behavior can trigger employee positive emotions and self-cognition, which may improve the performance of employees in innovation (Shih and Wijaya, 2017). Employees with excess qualifications are important training and guidance resources, they can use their previous knowledge and experience to identify problems to assist their peers better completion (Sikora et al., 2016). In addition, overqualified employees may use their skills for others, which is often considered as a potential advantage of hiring them (Erdogan et al., 2020). In other words, overqualified employees may engage in voice behavior toward peers, which will help them exhibit higher creative performance.

According to conservation of resources theory, individuals have the tendency to obtain, maintain, cultivate, and protect their cherished resources (Hobfoll, 1989). The perception that individual resources are wasted caused by overqualification may have a negative impact on the work attitude and behavior of employees (Arvan et al., 2019; Cheng et al., 2020). However, the conditional resources provided by environment and individual resources are not independent, but to jointly affect employees’ attitude and behavior (Hobfoll et al., 2018). Employees are embedded in the work team, investigating and assessing the behavior and performance of overqualified employees require consideration of their social environment (Jahantab et al., 2021). Peer group perceived overqualification represents the degree of similarity between overqualified employees and their peers (Hu et al., 2015). Similar qualifications to peers may enhance the sense of belonging of overqualified employees to the team, so as to encourage them to actively interact with peers and actively put forward suggestions that can improve peers’ work (Chu, 2021). Therefore, we predict that perceived overqualification will interact with peer group perceived overqualification to determine employee voice behavior toward peers.

Taken together, the current study seeks to make three contributions to the literature. First, we attempt to supplement and enrich the research on the positive results of overqualification by revealing that overqualified employees may show higher creative performance. Second, the few studies on the positive outcomes of overqualification mainly capture the perspective of person–environment (P–E) fit theory and self-representation theory (Li et al., 2019; Erdogan et al., 2020). Based on the conservation of resources theory, this study will provide a new perspective and ideas for understanding the positive results of overqualification by taking voice toward peers as an explanation mechanism for the impact of perceived overqualification on creative performance. Finally, previous studies mainly focused on the individual experience of overqualified employees (Dar and Rahman, 2020; Lee et al., 2021; Schreurs et al., 2021), largely ignoring the impact of team members on the overqualified employees’ attitude and behavior. This study expands the research of overqualification by taking peer group perceived overqualification as the boundary condition.

## Theory and Hypotheses

### The Moderating Role of Peer Group Perceived Overqualification on Perceived Overqualification and Voice Toward Peers

The conservation of resources theory (Hobfoll et al., 2018) holds that individuals have the tendency to preserve, protect and obtain the resources they think valuable, such as material, time, and qualification. Whether the threat of potential resource loss or the actual resource loss will lead to the avoidance behavior of employees. In addition, the conservation of resources theory also proposes that the situation of resource loss will enlarge the value of resources, and the resources obtained during resource loss have greater positive kinetic energy. Overqualification means that employees’ knowledge and skills are not fully utilized, which makes employees feel and recognize that their rich human capital is wasted, and the subsequent negative emotions will consume a lot of mental resources of overqualified employees (Lobene and Meade, 2013; Maynard et al., 2015; Erdogan et al., 2018). Similar to team members can supplement critical psychological resources for individuals, such as a sense of belonging (Chu, 2021). Depending on conservation of resources theory, overqualified employees who obtain resources may take positive behavior. Consequently, we believe that peer group perceived overqualification will affect the relationship between perceived overqualification and voice toward peers.

When the level of peer group perceived overqualification is high, overqualified employees may think they are a member of the team, resulting in a sense of belonging and trust in the work team (Chu, 2021), which increases employees’ emotional and social resources. The increase of supportive social resources enables overqualified employees more willing to exchange work-related professional knowledge and experience with peers and put forward constructive opinions on peers’ work (Shih and Wijaya, 2017). In addition, the similar level of qualification between overqualified employees and peers may become an adhesive to encourage them to appreciate each other (Hu et al., 2015). The harmonious atmosphere within the team makes overqualified employees feel that giving advice to peers will not be misunderstood and rejected by peers, and their opinions will be valued even if there are differences of opinion (Shih and Wijaya, 2017).

On the contrary, if an employee feels that he or she is one of the few overqualified people in the group, the focal employee may stay away from their peers and experience more negative emotions (Li et al., 2022). Putting forward suggestions and trying to change the status quo are social risks, which require time and energy (Detert and Burris, 2007). Therefore, overqualified employees will protect their existing social capital and prevent further loss of resources by inhibiting their voice behavior toward peers. Moreover, the difference between overqualified employees and their peers lead to nervousness of interpersonal relationships in the team (Sierra, 2011). The low trust and low emotional connection among team members in a tense atmosphere may cause overqualified employees to give up their voice toward peers for fear of causing conflicts with peers and damaging their social support resources (Shih and Wijaya, 2017). Based on the above arguments, we propose the following hypothesis.

*H1*: Peer group perceived overqualification moderates the relationship between perceived overqualification and voice toward peers, such that this link is positive (vs. negative) when peer group perceived overqualification is high (*vs.* low).

### Voice Toward Peers and Creative Performance

The conservation of resources theory may be particularly useful in explaining how voice toward peers is related to the implementation of new ideas and creative performance (Ng and Feldman, 2012). Various resources are needed in the creative process, including psychological and cognitive resources, and rich resources can make individuals more creative (Shalley and Gilson, 2004).

From the perspective of cognitive resources, employees involved in problem identification have a more accurate understanding of work, so they can put forward more useful and unique ideas (Yang et al., 2021). Besides, employees can get feedback on their opinions from other people in the team, and obtain additional information and knowledge by speaking out (Shih and Wijaya, 2017), so then further promote the generation of divergent thinking in the process of creative cognition.

From the perspective of psychological resources, voice is an indicator of good citizenship, employees who exhibit voice behavior toward peers are more likely to attract attention, get more appreciation, more respect, and higher evaluation from their peers, thus improving their confidence (Chen and Hou, 2016). Studies have shown that confident employees are more likely to generate new ideas (Steele et al., 2018). Furthermore, when employees express their views and concerns to peers, they are likely to experience positive emotions by truthfully speaking their views and acting according to their beliefs and values (Avey et al., 2012). Employees with positive emotions are more inclined to think creatively and divergently, thereby showing high creative performance (Song et al., 2017).

To summarize, voice toward peers can obtain the cognitive resources (information) and psychological resources (self-confidence and positive emotion) required for creative process, integrating and utilizing these resources will promote the creative performance of the speaker. Hence, we propose that voice toward peers has a positive impact on creative performance.

*H2*: Voice toward peers is positively related to creative performance.

Finally, in order to summarize the previous assumptions, we expect voice toward peers to mediate the relationship between perceived overqualification and creative performance. Perceived overqualification and peer group perceived overqualification should interact to affect voice toward peers. The degree to which they participate in discretionary actions that making constructive comments to peers should in turn relate to the individual’s creative performance. Perceived overqualification is expected to have positive indirect effects when peer group perceived overqualification is high and negative indirect effects when peer group perceived overqualification is low.

*H3*: Peer group perceived overqualification moderates the indirect effect of perceived overqualification on creative performance through voice toward peers, such that this indirect effect is positive (*vs*. negative) when peer group perceived overqualification is high (*vs*. low).

**Figure 1:**
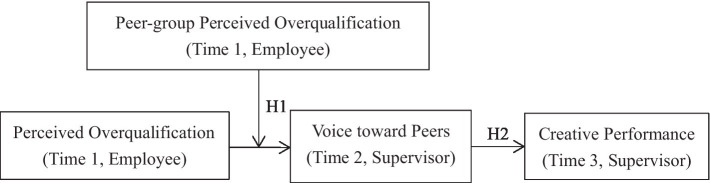
Study model.

The overall studied relationships are shown in Figure 1.

## Materials and Methods

### Sample and Procedure

We collected data at multiple times (T1, T2, and T3) by combining field survey and online questionnaire survey. Participants came from a manufacturing enterprise, a communication enterprise, and a financial enterprise in Shandong Province, China. We contacted the heads and human resources directors of these enterprises and introduced the purpose of the research, then obtained the support of them. Through preliminary interviews, we found that the recruitment requirements of these companies were relatively high in the industry, such as the level of education, but the salary has no obvious advantage. These characteristics make the participants suitable for our research.

Due to the uncertainty of employee access of computers, in order to ensure the collection of sufficient data, we used paper surveys to collect data at T1. We randomly invited 320 subordinates to fill in the questionnaires in the conference room in batches from the list of incumbents provided by the human resources department of each company. We assigned a unique number to each employee and recorded the names of them and their direct supervisors. We distributed the questionnaire by one-to-one correspondence between the questionnaire number and the employee number, and ensure that the correct number is listed in each survey to match the surveys at different times. We explained that the survey conclusions are only used for academic research and the survey results will be completely confidential to reassure the participants. More importantly, we encouraged employees and their direct supervisors to provide a valid e-mail address or WeChat ID (the popular Chinese mobile messenger app) and to actively participate in the second stage of online research. After each survey, we launched a raffle online and sent the link of the raffle to each participant’s e-mail or WeChat to increase the response rate. The winner of the raffle was eligible for one of three alternative prizes, which including a phone, tablet, or trendy shoes.

Three hundred employees filled out the survey that contained demographic characteristics and overqualification ratings at Time 1, with a response rate of 93.75%. In order to improve the efficiency of our research, we used an online survey website (Wenjuanxing, the Chinese version of Qualtrics) to conduct the survey 1 month later (T2). Drawing on previous studies on overqualification (Chu, 2021), we chose a 1 month interval to conduct the investigation to reduce the priming effects. Wenjuanxing generated a unique ID for each questionnaire, which was used along with the participant’s work number and e-mail address to match the online questionnaire to the paper questionnaire. Then, we emailed the link of the questionnaire to the immediate supervisors of the employees who completed the first survey and provided a list of names that matched the code for them to rate. After receiving the questionnaire, 61 supervisors rated voice behavior toward peers of 238 employees. One month later (T3), the 54 supervisors were asked to complete the third phase of the investigation using the same channels as T2. Then, a total of 54 supervisors rated the creative performance of 207 subordinates. Our final sample consisted of 206 subordinates and 53 supervisors after removing invalid dyads, constituting an overall response rate of 68.67%. Of the 206 subordinates, 164 (79.6%) were male. On average, subordinates were 33.46 years of age (*SD* = 5.21) and the working time with their supervisor was 19.44 months (*SD* = 17.72). A total of 199(96.6%) subordinates reported that they had a junior college degree or above.

### Measures

The subordinates’ voice toward peers and creative performance were rated by the direct supervisors, while the perceived overqualification was rated by subordinates. The English scale was translated into Chinese by using the back translation procedure recommended by Brislin (1986). All measures were anchored on a five-point Likert scale ranging from 1 = strongly disagree to 5 = strongly agree.

#### Perceived Overqualification

Perceived overqualification was assessed using the nine-item scale developed by Maynard et al. (2006). It measures the perceived overqualification in terms of knowledge, education, and abilities. Sample items are: “I have more abilities than I need in order to do my job” and “I have a lot of knowledge that I do not need in order to do my job.” Cronbach’s alpha was 0.84.

#### Voice Toward Peers

We adopted six-item scale developed by Van Dyne and LePine (1998) ^1^ and followed previous research to ask supervisors to evaluate their subordinates’ voice behavior toward peers (Erdogan et al., 2020). Sample items are: “This person communicates his or her opinions about work issues to colleagues even if his or her opinion is different, and colleagues disagree with him or her” and “This person gives constructive suggestions to colleagues to improve their work.” Cronbach’s alpha was 0.92.

#### Peer Group Perceived Overqualification

Peer group perceived overqualification was obtained by averaging the scores of all members of the same peer group on the perceived overqualification, after excluding the focal employee’s own score. The procedure of computing the average score after excluding the focal employee’s score provides an estimate of work group scores, which are not contaminated by common method variance problems caused by the use of self-reporting (Glomb and Liao, 2003). An existing study has shown that focal employees have the ability to detect the level of perceived overqualification reported by their peers (Hu et al., 2015), which provides support for us to use the average of perceived overqualification scores reported by peers to measure peer over qualification.

#### Creative Performance

Creative performance was evaluated by supervisors with five items adapted from the scale of George and Zhou (2002) scale. A sample items is: “This person comes up with new and practical ideas to improve performance.” Cronbach’s alpha was 0.90.

#### Control Variables

Following previous research on creative performance (Mohammed and Kamalanabhan, 2019; Wadei et al., 2021), we controlled for employee gender, age, education, and job tenure to avoid possible confounding effects. We also repeated the analyses while controlling these demographic characteristics. The results showed that there was no significant difference between the results with and without these controls. Therefore, for the sake of parsimony, we report the results without any of the controls.

## Results

### Confirmatory Factor Analysis

Before testing hypotheses, we used Mplus7.4 for confirmatory factor analysis (CFA) to evaluate the distinctiveness between variables: perceived overqualification, voice toward peers, and creative performance. The ratio of sample size to parameters should be exceed 10:1, otherwise, incorporating all scale items into the model will lead to some parameter estimation bias (Bandalos, 2002). Therefore, we packaged the scale items to form latent factors according to the high-estimate package strategy following Rogers and Schmitt (2004). According to the results (As shown in Table 1), the three-factor model was better than several alternative models in CFA (*χ*2 = 81.01; df = 41; RMSEA = 0.07; TLI = 0.96; CFI = 0.97), showed sufficient overall fit and high discriminative validity.

**Table 1:** Model fit results for confirmatory factor analyses.

Model	*χ*2	df	*χ*2/df	TLI	CFI	RMSEA	SRMR	∆*χ*2
Three-factor model	81.01	41	1.98	0.96	0.97	0.07	0.04	
Two-factor model	395.45	46	8.60	0.67	0.72	0.19	0.22	314.44
Single-factor model	971.28	52	18.68	0.22	0.27	0.29	0.32	890.27

*Three-factor model: conceptual model. Two-factor model: perceived overqualification and voice toward peers combined, Single-factor model: all variables combined. CFI is the comparative fit index. RMSEA is the root-mean-square error of approximation. SRMR is the standardized root-mean-square residual. TLI is the Tucker–Lewis index.*

### Tests of Hypotheses

The means, standard deviations, correlations between the variables and internal consistency coefficient of the scale are showed in Table 2. As shown in Table 2, voice toward peers was positively related to creative performance (*r* = 0.52, *p* < 0.01), which provides preliminary statistical support for the following hypothesis 2.

**Table 2:** Means, standard deviations, correlations, and internal consistency coefficient.

	*M*	*SD*	1	2	3	4	5	6	7	8
1. Age	33.47	5.21								
2. Gender	1.20	0.40	−0.38^**^							
3. Education	3.86	0.67	0.08	0.13						
4. Job tenure	19.44	17.72	0.16^*^	−0.10	0.01					
5. POQ	2.85	0.61	−0.02	0.05	−0.04	−0.02	(0.84)			
6. Voice toward peers	3.51	0.78	−0.17^*^	0.06	−0.03	0.00	−0.12	(0.92)		
7. Peer group poq	2.84	0.40	−0.07	0.24^**^	−0.06	−0.05	0.17^*^	−0.11		
8. Creative performance	3.18	0.82	−0.11	−0.05	−0.04	0.05	−0.05	0.52^**^	−0.04	(0.90)

*N = 206. gender: 1 = male, 2 = female; education: 1 = junior middle school, 2 = high school, 3 = junior college, 4 = bachelor, 5 = master, 6 = doctor. POQ = perceived over qualification.*

* *p* < 0.05;

** *p* < 0.01.

**Figure 2:**
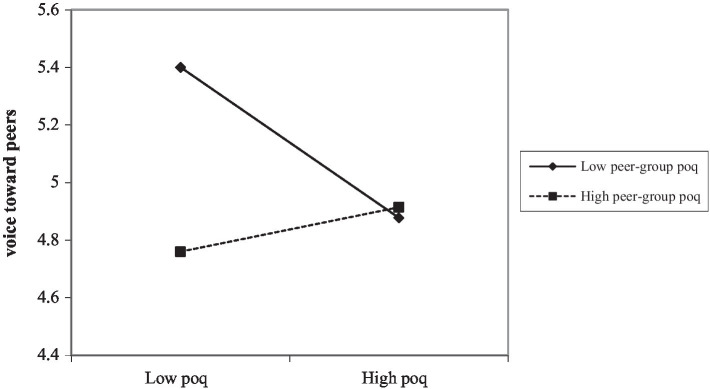
The interaction between perceived overqualification and peer group perceived overqualification on voice toward peers.

We used SPSS 22.0 for regression analysis to test H1 and H2. Hypothesis 1 predicted that peer group perceived overqualification would moderate the relationship between perceived overqualification and voice toward peers. The results presented in Table 3 showed that the interaction between perceived overqualification and peer group perceived overqualification was a significant predictor of voice toward peers (Model 3, *β* = 0.70, *p* < 0.01). In order to demonstrate the nature of the moderating effect more clearly and intuitively, we plot the interaction by calculating the slope of one standard deviation above and below the mean of peer group perceived overqualification. As indicated in Figure 2, perceived overqualification was positively related to voice toward peers when peer group perceived overqualification at high level, and negatively related to voice toward peers when peer group perceived overqualification at low level.

**Table 3:** Results of regression analysis.

Outcome	Voice toward peers			Creative performance	
	Model1	Model2	Model3	Model4	Model5
	Perceived overqualification	−0.15	−0.13	−0.15	
0.01		Peer group Perceived overqualification		−0.17	−0.38^*^
	0.023		Interaction		
0.70^**^				Voice toward peers	
		0.55^***^	0.55^***^		*R* ^2^
0.01	0.02	0.07	0.27	0.27	
△*R*^2^		0.01	0.05^**^		0.00
△*F*	2.81	1.55	9.57^**^	76.44^***^	74.68^***^

* *p < 0.05;*

** *p < 0.01;*

*** *p < 0.001*.

Therefore, hypothesis 1 was supported.

Hypothesis 2 predicted that voice toward peers is positively related to creative performance. As shown in Table 3, voice toward peers was found to have a positive correlation with creative performance (Model 5, *β* = 0.55, *p* < 0.001). Hence, hypothesis 2 was supported.

We used the analysis method recommended by Preacher et al. (2007) to estimate the indirect effects of non-normal distribution. 95% confidence intervals were calculated to test the moderated indirect effects by using 5,000 repeated parameter sampling. Table 4 revealed that when peer group perceived overqualification at high level, the relationship between perceived overqualification and creative performance through voice toward peers was not significantly positive [*β* = 0.07, 95% confidence interval (−0.03, 0.20)]; however, when peer group perceived overqualification at low level, the relationship between perceived overqualification and creative performance through voice toward peers was significantly negative [*β* = −0.24, 95% confidence interval (−0.41, −0.10), excluding 0]. The difference between the two groups was significant, and the 95% confidence interval [0.13, 0.53], excluding 0, showed that the indirect effects are certainly different when peer group perceived overqualification at different level. Consequently, Hypothesis 3 was supported.

**Table 4:** Results of moderated mediation effect.

	*β*	SE	95% confidence interval	
			Lower limit	Upper limit
Conditional indirect effect				
High peer group poq (+1 *SD*)	0.07	0.06	−0.03	0.20
Low peer group poq (−1 *SD*)	−0.24	0.08	−0.41	−0.10
Diff	0.31	0.10	0.13	0.53

*High and low refer to one standard deviation above and below the mean value of peer group perceived overqualification. β and SE refer to the unstandardized parameter estimates and their corresponding standard errors, respectively*.

## Discussion

Based on the conservation of resource theory, we constructed a theoretical model of perceived overqualification and creative performance. We further explore the mediating role of voice toward peers on the relationship between perceived overqualification and creative performance, as well as the moderating role of perceived overqualification in influencing the mediation.

Using data from employees in China gathered across three time periods from three enterprises, we found support for our model. The results revealed that peer group perceived overqualification moderated the relationship between perceived overqualification and voice toward peers. Interestingly, perceived overqualification negatively affected voice toward peers when peer group perceived overqualification at low level and positively affected voice toward peers when peer group perceived overqualification at high level. Furthermore, we find that peer group perceived overqualification moderated the indirect effects of perceived overqualification on creative performance *via* voice toward peers. Notably, the indirect effect was negative only when peer group perceived overqualification at low level and became insignificant when peer group perceived overqualification at high level.

### Theoretical Implications

First, we promote the research of overqualification theoretically and empirically by identifying creative performance as the potential positive outcome of overqualification. The existing literature on overqualification gives a large number of negative descriptions of overqualified employees, because the field has mainly relied on equity theory and relative deprivation theory (Cheng et al., 2020; Schreurs et al., 2021). In fact, overqualified employees have more knowledge and skills, which may be beneficial to the organization (Sikora et al., 2016). However, the research on perceived overqualification and positive outcomes has been largely ignored. Moreover, the relationship between overqualification and employees’ task performance is inconsistent (Li et al., 2019; Lee et al., 2021). This study can theoretically explain the inconsistent results to a certain extent by exploring the influence mechanism of perceived overqualification on creative performance from the perspective of resources.

In addition, this study deepens the understanding of the positive results of overqualification and provides a new perspective and new ideas for future research. Although a few studies have explored the possible positive effects of overqualification based on person–environment (P–E) fit theory and self-representation theory (Li et al., 2019; Erdogan et al., 2020), to some extent, they ignore the process of obtaining resources in the interaction between individuals and peers. However, we find that resources are an indispensable key factor involved in creative processes (Shalley and Gilson, 2004). Therefore, based on the conservation of resource theory, the present study introduces voice toward peers as an interpretation mechanism between perceived overqualification and creative performance, which provides a valuable supplement to the literature. At the same time, we respond to the call to explore the theoretical mechanism behind the relationship between overqualification and creativity (Luksyte and Spitzmueller, 2016).

Another noteworthy implication is that our study further identifies the peer group perceived overqualification as an important boundary condition of overqualification–creative performance. Previous research has revealed overqualification to be both dysfunctional (Simon et al., 2019) and functional (Zhang et al., 2016). However, these studies did not consider the membership of the staff in the team, nor did they incorporate the team background into the research model. We extend the existing literature by exploring how peer group perceived overqualification affects the voice behavior of overqualified employees to their peers. As our empirical results show that peer group perceived overqualification changed the negative effect of perceived overqualification on voice toward peers, indicating that contextual factors can be implemented to mitigate the negative consequences of overqualification. Furthermore, the findings of the research contribute to theory building of perceived overqualification through distinguishing perceived overqualification as an individual characteristic from the perception of overqualification that occurs in teams.

### Practical Implications

In addition to the theoretical and empirical contributions discussed above, we offer several practical implications for both organizational management and human resource practices. The higher productivity and possible knowledge spillover effects of overqualified employee offset the cost of employment (Jones et al., 2009; Pouliakas, 2013), and such hiring decision may bring benefits in the form of voice behavior toward peers and creative performance if managed appropriately, although it may be associated with some risks. Our results reveal that perceived overqualification positively affects creative performance through voice toward peers when peer group perceived overqualification at high level. Therefore, managers should keep in mind that need not deliberately screen out such applicants when recruiting employees, but to pay more attention to overqualified employees, because they tend to have more favorable reactions toward peers and make more contributions to organization when they working with similarly overqualified peers. Managers should be aware that they can promote overqualified employees’ voice behavior toward peers and creative performance if they strive to strategically position overqualified employees in the organization and group these similar employees several overqualified employees into a team. Further, managers also should encourage overqualified individuals to cooperate actively with their peers and benefit others to build team spirit. Finally, managers should carry out some activities outside the workplace to strengthen the communication between overqualified employees and their peers, so as to enable them to establish a good interactive relationship. By doing so, overqualified employees can apply their underutilized qualifications to support the group and improve their creative performance by demonstrating voice behavior toward peers.

### Limitations and Future Research

Like any other study, the present study has some limitations should be noted. First, although the examined variables were measured at three periods, the design of time-lagged still cannot set up causality explicitly for all paths in our model. We recommend that future studies to extend our study using longitudinal designs or experiments to rule out the reverse causality in a more rigorous manner.

Second, although we controlled for employee age, gender, education, and job tenure, the results have no significant difference with and without control variables. Future research can take other factors that may affect voice behavior and creative performance as control variables, such as the exchange quality of leader–members and organizational identification (Olsson et al., 2012; Wang et al., 2018).

Third, although our study examined a mechanism links perceived overqualification with creative performance in which voice toward peers as a mediator and peer group perceived overqualification as a boundary condition, other factors may affect the relationship we examined. More research is needed to examine other potential moderators to reduce the negative effects of hiring overqualified employees, for example, empowering leadership may help the overqualified employees to establish a more positive psychological state, give them full opportunities for growth, and provide needed help and guidance to promote the overqualified employees to exert their redundant qualifications (Hon and Chan, 2013).

Finally, the data of 53 groups from different industries are used in this study and the level of excess qualification and creative forms may be different. Therefore, the conclusion of this study can be further verified for a certain industry or a certain type of employees in the future. In addition, the study was conducted in China with collective cultural characteristics (Hofstede, 2001), further cross-cultural research is needed to explore whether the results of this study are applicable in other cultural backgrounds. To extend our finding, future studies should consider variables at the national level, explore the generalizability of our results in different countries which have different economic conditions, different educational level, and different universality of overqualification.

## Conclusion

In this study, we based on conservation of resources theory to examine the moderating effect of peer group perceived overqualification and mediating effect of voice toward peers on the relationship between perceived overqualification and creative performance by using a Chinese sample and also clarify important implications for future research and practice. A reasonable extension of our research results is that managers can stimulate employees’ voice behavior toward peers and improve their creative performance by pairing overqualified employees. Such effort is worthwhile, both for the individuals and for the organization.

## Data Availability Statement

The raw data supporting the conclusions of this article will be made available by the authors, without undue reservation.

## Ethics Statement

The studies involving human participants were reviewed and approved by Institutional Review Board of Shanghai University, China. Written informed consent for participation was not required for this study in accordance with the national legislation and the institutional requirements.

## Author Contributions

YiL, YanL, PY, and MZ designed the study and revised the draft. YiL and PY collected the data. YanL drafted the theory and results. MZ drafted the methods. All authors contributed to the article and approved the submitted version.

## Funding

This research was funded by the municipal general program of Shanghai educational science research (Project No. C2022012).

## Conflict of Interest

The authors declare that the research was conducted in the absence of any commercial or financial relationships that could be construed as a potential conflict of interest.

## Publisher’s Note

All claims expressed in this article are solely those of the authors and do not necessarily represent those of their affiliated organizations, or those of the publisher, the editors and the reviewers. Any product that may be evaluated in this article, or claim that may be made by its manufacturer, is not guaranteed or endorsed by the publisher.

## Footnotes

^1^First, when controlling for employee age, gender, education and job tenure, the regression analysis results showed that the relationship between the interaction of perceived overqualification and peer-group perceived overqualification and voice toward peers was significantly positive (*β* = 0.60, *p* < 0.05); The relationship between the interaction of perceived overqualification and peer-group perceived overqualification and voice toward peers was still significantly positive (*β* = 0.70, *p* < 0.01) without any of the controls. Second, the relationship between voice toward peers and creative performance was significantly positively with (*β* = 0.55, *p* < 0.001) and without control variables (*β* = 0.55, *p* < 0.001). Finally, we found that the 95% confidence interval between the groups to test the moderated mediation effect with (*β* = 0.55, *p* < 0.001) and without control variables (*β* = 0.55, *p* < 0.001) both exclude 0 and were significant.
